# Gas pressure evolution characteristics of deep true triaxial coal and gas outburst based on acoustic emission monitoring

**DOI:** 10.1038/s41598-022-26288-7

**Published:** 2022-12-16

**Authors:** Xin Zhang, Jupeng Tang, Honghao Yu, Yishan Pan

**Affiliations:** 1 School of Mechanics and Engineering, Liaoning Technical University, No. 47, Zhonghua Road, Xihe District, Fuxin, 123000 Liaoning China; 2 School of Environment, Liaoning University, Shenyang, 110036 Liaoning China

**Keywords:** Natural hazards, Solid Earth sciences

## Abstract

Coal and gas outburst is one of the geological disasters that seriously threaten the safety of coal mines production. In recent years, with the increase of mining depth, outbursts become frequent. To further explore the occurrence mechanism of deep coal and gas outburst, a self-developed true triaxial coal and gas outburst simulation device was used to simulate the coal and gas outburst at different depths. The results show that with the increase of simulation depth, the critical gas pressure of outburst gradually decreases, and the unit outburst intensity increases sharply. The gas threshold of deep coal and gas outburst is lower. During the incubation and excitation, gas pressure has three special variation rules, namely self-increasing characteristic, stage and instantaneous. In the early incubation, acoustic emission (AE) energy is at a low level, low energy frequency is dominant; in the later incubation, AE energy increases greatly, high energy frequency is dominant. From the perspective of AE energy, a quantitative index that reflects the danger of coal and gas outburst in the incubation is defined, which provides a scientific reference for the prediction and prevention of disasters of coal and gas outburst in deep mining.

## Introduction

A coal and gas outburst (hereafter referred to as an outburst) is an extremely complex mine gas dynamic phenomenon in coal mine production, characterized by the sudden throwing of a large amount of coal and gas into the roadway or stope in a very short time, which poses a seriously threat to the lives of miners, facilities, and coal production^1^. Since the first outburst accident reported in France in 1834, more than 40,000 outburst-related accidents have been recorded around the world and their intensity and frequency tend to increase with the increasing depth of mining^2–4^. Consequently, the mechanisms of outburst have been the subject of research in major coal producing countries^5,6^. However, China is the largest producer and consumer of coal in the world, and effective prediction and prevention of outburst disasters remains a huge challenge for the Chinese government^7^.

Owing to the sudden, dangerous, and hidden threat of outbursts, it is difficult to study the outburst mechanism directly on site. Therefore, laboratory testing had been conducted by many scholars to study the outburst phenomenon and mechanism. A laboratory outburst simulation experiment was firstly conducted in 1953^8^. Over the past few decades, suitable outburst-testing equipment has been developed, which is greatly promoting the realization of large-scale physical experiments^9–12^. Yin et al.^13^ developed an apparatus with size of 570 mm × 320 mm × 385 mm. The device can accommodate large coal samples with different coal seam dip angles and outburst intensity under different geological stresses and gas pressures. Cao et al.^14^ developed a novel large-scale three-dimensional apparatus to study mechanisms of coal and gas outburst with a size of 1500 mm × 800 mm × 800 mm. The outburst port can be initiated actively and instantaneously. Lu et al.^15^ developed a multi-functional physical simulation device for dynamic disasters in deep mines. Model dimensions are 1200 mm × 1200 mm × 2000 mm with a maximum triaxial stress of 10 MPa. It provides the possibility to simulate the occurrence conditions of high-stress gas coupling coal seams and the simulation of deep underground excavation. Yuan^16^ developed a large-scale outburst simulation experimental platform with a model size of 3300 mm × 3250 mm × 4100 mm and maximum boundary stress of 5 MPa.

Generally, the outburst is generalized into the characteristic comprehensive action of stress, gas, and coal^17–19^. Yin et al.^20^ investigated the combined effects of stress, pore pressure and temperature on methane permeability. Pan et al.^21^ quantitatively investigated the damage characteristics and outburst mechanism of tectonic raw coal under different gas pressure and stress conditions. The experimental results show that the damage to coal samples increases with the increase in gas pressure. However, many scholars' studies have shown that the main source of outburst energy is the gas in the coal seam. Halbaum^22^ proposed the theory that gas plays a dominant role in outburst. It believes that the danger of outburst is related to the gas pressure gradient in front of the working face, the steeper the gas pressure curve, the easier it is to outburst. Dai et al.^23^ determined that the main energy source of outbursts originated from the elastic potential energy of the coal body and the internal energy of the gas. Valliappan^17^ believes that the release of gas energy due to desorption of methane from the coal matrix to the pores is the major cause for the occurrence of outbursts. Guo et al.^24^ explored the variation characteristics of gas pressure during an outburst under different initial gas pressures and coal quantities.

Based on AE signals real-time, and continuous monitoring of the stress state and fracturing process of rock can be realized^25^. Li et al.^26^ used self-developed AE continuous monitoring and early warning equipment to monitor and predict the dynamic disasters of outburst in the working face. It was found that before the outburst, AE had an obvious increasing trend before the gas concentration change. Zhao et al.^27^ proposed that the total events, large events and energy parameters of AE can better reflect the characteristics of AE activities and that the increase in total events and the sharp increase in major events are signs of outburst. He et al.^28^analyzed the coupling relationship between damage and AE of coal and rock mass and discussed the feasibility of using AE to evaluate the risk of rock burst. Lu et al.^29^ and Li et al.^30^ discussed the mechanical behaviors and acoustic emission fractal characteristics of coal specimens under compression.

As discussed, outburst is a mechanical process of coal body damage and energy accumulation. Gas is the main energy source of outburst, the energy provided by gases is one to two orders of magnitude larger than that provided by the in-situ stress^17^. Therefore, the evolution of gas before outburst can reflect the outburst intensity to some extent. Regarding the current situation of coal mining in China, with its increasing depth, the corresponding geo-stress, gas pressure, and content have also gradually increased^31,32^. The existing research on its evolution mainly focuses on the post-outburst process. For example, Peng et al.^33^ carried out outburst simulation tests and obtained the evolution process of gas pressure in coal after the outburst started and its response to different in situ stress conditions.

Aiming at the shortcomings of existing research, this paper has made a breakthrough from the direction of outburst simulation tests. Based on the self-developed true triaxial coal and gas outburst physical simulation test system, five groups of outburst simulation tests with different depths were carried out, and the four stages of outburst incubation, excitation, development, and termination were completely reproduced. The evolution and precursor characteristics of gas pressure and AE energy were monitored. The connection between the gas pressure and the acoustic emission signal during the outburst is established. Additionally, from the perspective of AE energy, a quantitative index reflecting the danger of outburst in the incubation stage is defined. This work provides a scientific reference for the prediction and early warning of dynamic disasters in deep mining coal and gas outbursts.

## Experimental

### Sample preparation

The test coal samples were selected from the outburst coal seam of the Sunjiawan Mine in Fuxin, Liaoning Province, northern China. The coal seam is a medium-rich ash long flame coal with a gas content of 15.1 m^3^/t, an apparent coal density of 1.52 g/cm^3^, a true density of 1.593 g/cm^3^, a compressive strength of 14.42–31.28 MPa, and a porosity of 6.2–11.3%. Considering the difficulty of obtaining large-scale raw coal samples, briquette samples were used in this study. Studies have shown that the coal briquette obtained by crushing non-structural coal blocks into a certain particle size and pressing through proportioning exhibited similar mechanical strength, adsorption and desorption properties to those of the field tectonic coal masses^16,34–37^. The average moisture content of different particle size ranges is 3.7%. To make briquettes bond better, a part of water is added to make the moisture content of the briquette reach 5%, and the well-proportioned coal powder is sealed and stored for the test. Although the adsorption capacity of coal for methane (CH_4_) and nitrogen (N_2_) and the strain difference induced by adsorption are different, the adsorption mechanism of coal for these two gases is the same^38^. Owing to a large quantity of gas sources being needed and considering laboratory experimental safety, N_2_ was selected instead of CH_4_ for testing^39^.

### Experimental device

Physical simulation experiment can reflect its physical nature to the greatest extent, and the success of similarity simulation experiment must be based on the similarity theory. As one of the key parameters to determine the size of the experimental device, the geometric similarity coefficient must be selected reasonably. Larger size can better eliminate the impact of size effect and boundary effect on the outburst, and the similarity degree is higher. However, the loading capacity and sealing capacity of the system are required, and the preparation of coal sample costs too much manpower and material resources. If the size is small, it will be greatly affected by the size and boundary effect, and the similarity coefficient cannot be changed, and the flexibility is small, but it has the advantages of simple operation, easy realization, and small cost.

**Figure 1:**
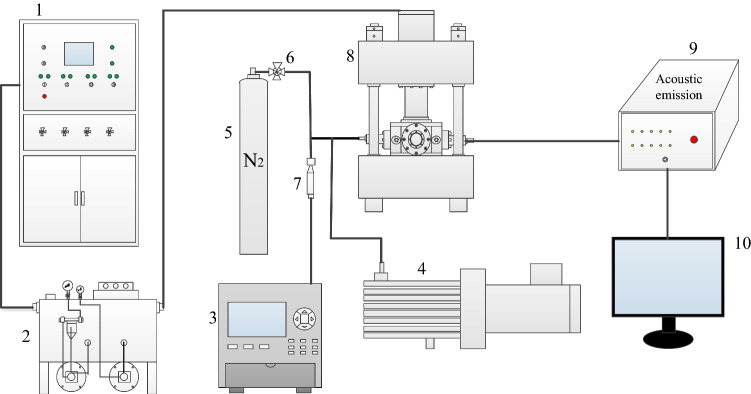
True triaxial coal and gas outburst physical simulation test system: 1—console; 2—hydraulic station; 3—data collector; 4—vacuum pump; 5—gas cylinder; 6—valve; 7—gas pressure sensor; 8—true triaxial tester; 9—acoustic emission; 10—acoustic emission data acquisition system.

In this test, a self-developed true triaxial coal and gas outburst physical simulation test system is used, as shown in Fig. 1. The test system is mainly composed of in-situ stress loading system, hydraulic control system, gas pressure loading system, acoustic emission monitoring system and data acquisition system.

**Figure 2:**
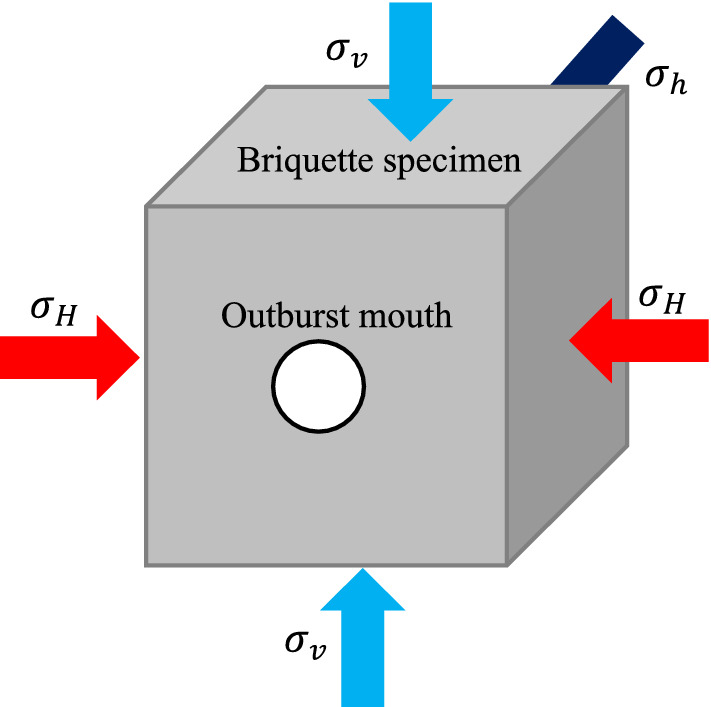
Schematic diagram of true triaxial mechanics.

The main structure of the equipment is mainly composed of 200 t upper end press, stress chamber and base. The internal cavity of the experimental system, which is where the pulverized coal is pressed, measures 200 mm × 200 mm × 200 mm. The pulverized coal is directly pressed into shape by the upper end press, and the maximum molding pressure can reach 30 MPa. Through the three-dimensional independent loading and unloading system, the true triaxial stress loading of the test specimen can be realized (Fig. 2), the maximum loading pressure is 25 MPa, and the cylinder loading stroke is 20 mm. There are 24 gas injection ports and various sensor interfaces on the 4 side of the model, which can measure the changes of stress, temperature, and acoustic and electrical signals in the model cavity. The front of the model is provided with a circular coal and gas outburst mouth with a diameter of 80 mm, and a plexiglass baffle is installed at the outburst mouth to simulate the weak surface. The plexiglass is fixed and sealed with a plate and sealing rubber ring to induce coal and gas outburst^40^.

### Experimental design

Li et al.^41^ collected data from more than 600 mining areas in mainland China, and plotted the distribution of the deep underground stress with respect to depth, shown as Eq. (1). Based on previous experience^42^, the density ratio of raw coal to briquette can be approximately 1.1, length ratio 7.5, in-situ stress similarity ratio 8.3, gas pressure similarity ratio 1.0. Then the test vertical stress, maximum horizontal stress, and minimum horizontal stress are determined. The specific stress and gas pressure loading scheme are shown in Table 1.

1 $$\begin{gathered} \sigma_{v} = 0.0208{\text{H}} + 2.195 \hfill \\ \sigma_{H} = 0.0238{\text{H}} + 7.648 \hfill \\ \sigma_{h} = 0.0184{\text{H}} + 0.948 \hfill \\ \end{gathered}$$

where *H* denotes depth (m); $\sigma_{v}$ is vertical in-situ stress (MPa); $\sigma_{H}$ is the maximum horizontal in-situ stress (MPa); $\sigma_{h}$ is the minimum horizontal in-situ stress (MPa).

**Table 1:** Experimental scheme of coal and gas outburst.

Simulation depth (m)	Actual in-situ stress (MPa)			Test stress (MPa)			Gas pressure (MPa)
	$\sigma_{{\text{H}}}$	$\sigma_{{\text{h}}}$	$\sigma_{{\text{v}}}$	$\sigma_{H}^{^{\prime}}$	${\upsigma }_{{\text{h}}}^{^{\prime}}$	${\upsigma }_{v}^{^{\prime}}$	
600	14.675	21.928	11.988	1.76	2.64	1.44	0.75–1.0 MPa, load and stabilize for 200 s;1.0–2.0 MPa, loading at 0.2 MPa per level and stabilize for 100 s; 2.0–3.0 MPa, loading at 0.1 MPa per level and stabilize for 100 s until outburst occurs
800	18.835	26.688	15.668	2.20	3.20	1.88	
1000	22.995	31.448	19.348	2.70	3.80	2.30	
1200	27.155	36.208	23.028	3.27	4.36	2.77	
1400	31.315	40.968	26.708	3.77	4.93	3.21	

**Figure 3:**
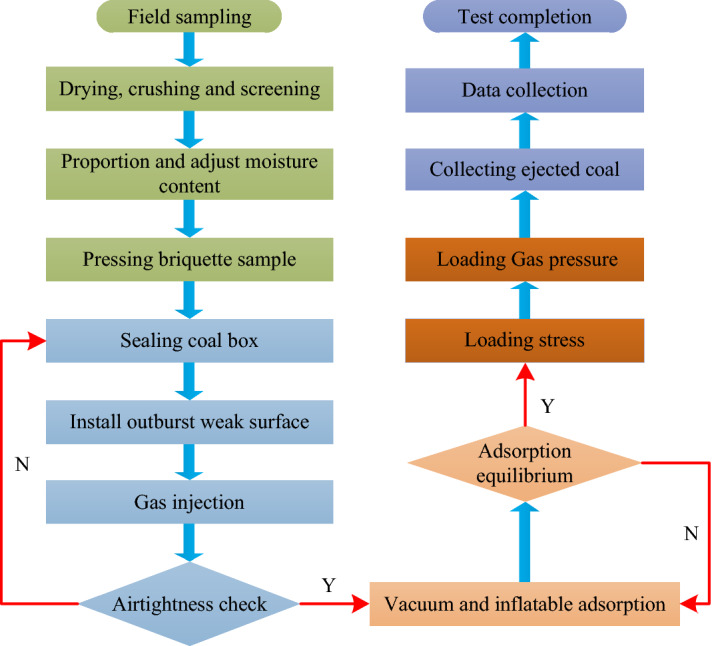
Flowchart of coal and gas outburst experiments.

The experimental procedures of outburst tests included the following main steps (Fig. 3).

1. Preparation of briquette samples: raw coal sampling → crushing → screening → adjust moisture content → proportioning^43^ → pressing briquette sample.
2. Airtightness check: after briquette pressing is completed, connect each operating system to check the airtightness of the coal box. If it is good, carry out the next test, otherwise continue to check the airtightness.
3. Vacuuming and adsorption: after the pressure chamber is evacuated for 1 h, nitrogen is injected to the adsorption equilibrium.
4. Stress and gas pressure loading. Based on stress similarity ratio, the value of the test stress is obtained. According to the Specification of Coal and Gas Outburst Prevention in China, the critical gas pressure is 0.74 MPa. So, the minimum gas pressure is set to 0.75 MPa. The specific stress and gas pressure loading scheme are shown in Table 1.
5. Data collection: the coal powder after outburst will be collected by region, and the data collected by pressure acquisition collector and acoustic emission system will be saved.
6. Repeated test: repeat the above steps, change the stress loading conditions, carry out the next set of tests, and compare and analyze the obtained results.

### Experimental phenomenon

**Figure 4:**
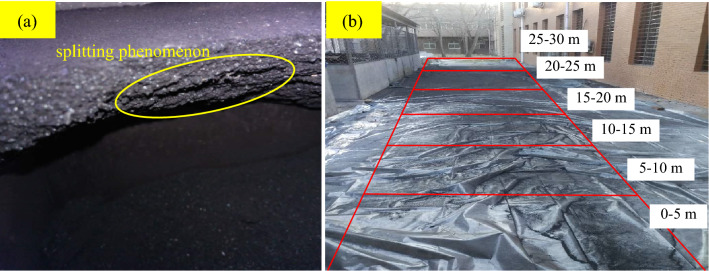
The characteristics of coal and gas outburst [(**a**). splitting phenomenon of outburst hole (**b**). Sketch of different areas of outburst coal powder].

**Figure 5:**
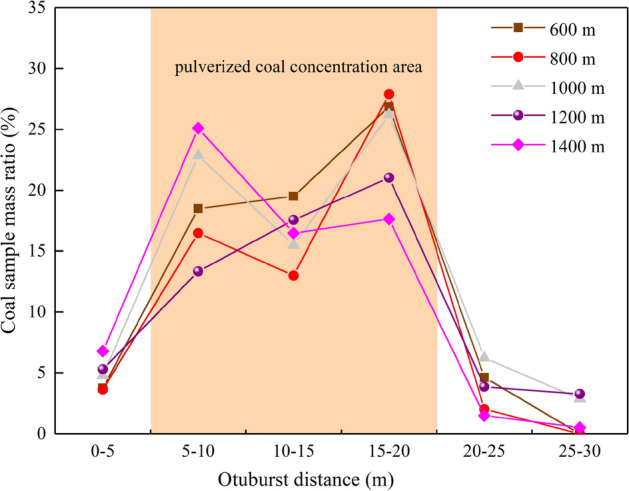
The mass ratio of pulverized coal under different simulation depths.

During the test, when the gas pressure was gradually loaded to the critical gas pressure, the gas carried by the coal body was violently ejected in trumpet shape, accompanied by a violent loud noise, and the outburst lasted for 0.6–1.8 s. The ejected coal and gas had a high initial velocity to form coal–gas storm flow. The test results are shown in Table 2. There are a lot of hand-twisted non-sensitive coal powder in the surface layer of the outburst distal end, and the outburst weak face hole has typical characteristics of small mouth and large cavity. It indicates that the initiation of coal and gas outburst is the result of sudden release on the weak surface of outburst when the energy accumulated by the gas-bearing coal body reaches the critical state. After outburst, the residual coal sample of outburst hole will appear "splitting phenomenon" (Fig. 4a), which is similar to the field observation result^44^. The coal powder after outburst is divided into 6 areas according to the distance from the outburst mouth: 0–5 m, 5–10 m, 10–15 m, 15–20 m, 20–25 m and 25–30 m, as shown in Fig. 4b. Figure 5 shows the proportion of coal powder in different areas.

**Table 2:** Experimental results of coal and gas outburst under different simulation depths.

Simulation depth (m)	Total coal mass (kg)	Absolute outburst intensity (kg)	Relative outburst intensity (%)	Critical gas pressure (MPa)	Unit outburst intensity (kg/MPa)	Outburst distance (m)
600	8.232	7.121	86.50	3.010	2.366	21.8
800	8.878	7.624	85.86	3.166	2.408	20.5
1000	8.562	7.037	82.19	2.554	2.755	26.3
1200	8.741	6.858	78.46	1.892	3.625	27.1
1400	8.858	6.543	73.86	1.638	3.995	26.5

The results show that the distribution of thrown pulverized coal has fluctuating characteristics, indicating the fluctuation of the outburst energy release and dissipation^45^. The mass distribution of the pulverized coal along the direction of the outburst under different simulation depths is basically the same, and the pulverized coal concentration area is 5–20 m. When outburst occurs, the coal samples initially injected have the largest energy, so the injection distance of this part of the coal sample is relatively far, mainly concentrated in 15–20 m, and the outburst coal has the highest mass. As the outburst goes on, the energy of outburst is obviously weaker than that of the first. Therefore, the injection distance of this part of coal sample is smaller than that of the first, mainly concentrated in 5–15 m. In 0–5 m, the mass of outburst coal is relatively small, and it can be concluded that the outburst energy will decay rapidly when the outburst is approaching the end. However, at 1400 m, the maximum coal sample mass is in 5–10 m. This is because the gas pressure that reaches the critical outburst condition is small and the outburst duration is short, resulting in insufficient release of outburst energy, and the outburst coal samples are accumulated near the outburst mouth.

## Results and analysis

Gas pressure is a necessary condition for the occurrence of coal and gas outbursts and is one of the main energy sources of outbursts. Its influence in the incubation and excitation process of outburst cannot be ignored. The gas existing in coal cracks and pores has three main effects on coal: (1) Under the action of gas pressure gradient, the gas adsorbed on the surface of coal micropores can penetrate the coal capillary pores. The permeability of adsorbed gas reduces the mechanical strength of coal body and is conducive to the occurrence of coal and gas outburst^34,46,47^; (2) The desorption of gas further strengthens the coal damage and crack propagation^48,49^; (3) Comprehensively compress the skeleton structure of the coal to promote the elastic potential of the coal body. Therefore, it is of great significance to study the evolution characteristics and precursor rules of gas pressure in the process of coal and gas outburst through laboratory experiments, which is one of the effective ways to study coal and gas outburst disasters.

### Analysis of influence factors of critical gas pressure

Gas pressure is the main dynamic to complete outburst and eject coal powder^50^, and its magnitude is directly related to the intensity and damage degree of coal and gas outburst. In this paper, the instantaneous outburst gas pressure value is defined as the critical gas pressure *P*_cr_, and the absolute outburst intensity under the critical gas pressure state is defined as the unit outburst intensity *I*_U_. Studying the relationship between simulation depth, critical gas pressure, and unit outburst intensity is helpful to deeply understand the mechanism of coal and gas outburst.

**Figure 6:**
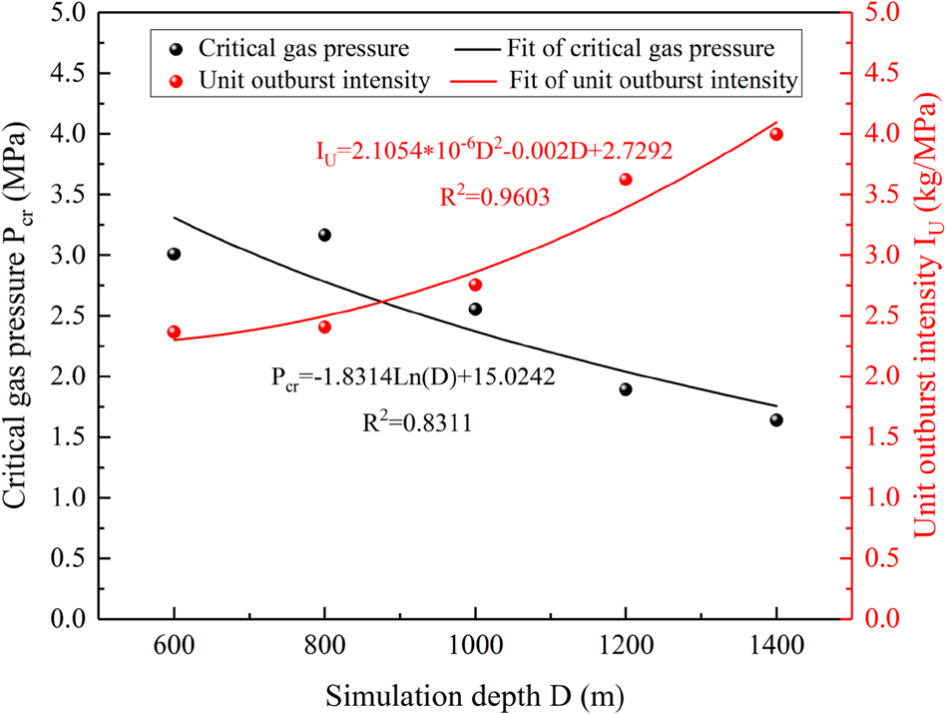
Variation of critical gas pressure and unit outburst intensity with simulation depth.

The critical gas pressure is logarithmically decreasing with the simulation depth, and the unit outburst intensity is quadratic increasing with the simulation depth. It can be seen from Fig. 6 that when the simulation depth increases from 600 to 800 m, the critical gas pressure increases by 5.18%, and when the simulation depth increases from 800 to 1000 m, 1200 m and 1400 m, the critical gas pressure decreases by 19.33%, 25.92%, and 13.42%, respectively. With the increase of simulation depth, the critical gas pressure shows a downward trend overall, and the decreasing range increases first and then decreases. The unit outburst intensity increases from 2.366 to 3.995 kg/MPa, it shows that the unit outburst intensity increases sharply with the increase of the simulation depth. This is because the in-situ stress of deep coal body increases gradually. Under the condition of high in-situ stress, the coal body has a high degree of damage, and it is easy to reach the critical condition and cause instability and outburst. Therefore, the critical gas pressure gradually decreases with the increase of simulation depth. It indicates that in the incubation, the in-situ stress is dominant, which causes deformation and failure of the coal body.

**Figure 7:**
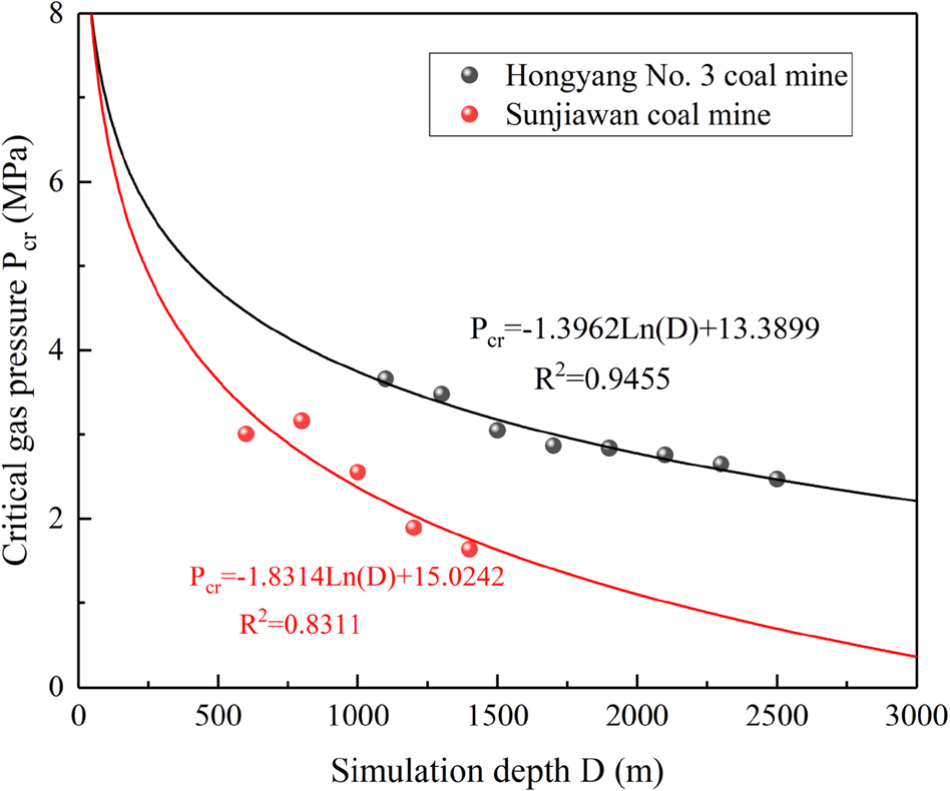
Comparative analysis of critical gas pressure for outburst.

Coal and gas outburst is a kind of geological disaster. According to the theory of comprehensive action hypothesis^12^, its occurrence is the result of the combined action of factors such as in-situ stress, gas, and coal properties. The test plan was designed based on Sunjiawan coal sample and coal seam parameters, and the results obtained have certain limitations. To explore the general variation rule of outburst critical gas pressure with depth, the test results were compared with those obtained in previous studies^40^, as shown in Fig. 7.

There are great differences in coal types and mechanical properties of coal in different areas due to their own formation and geological conditions. The 12th coal seam in Hongyang No. 3 Mine belongs to the outburst dangerous coal seam, with an average thickness of 1.89 m, mainly lean coal, and its compressive strength is between 6.94 and 9.04 MPa. The coal used in this test is sourced from the Sunjiawan Coal Mine, which is a high-gas coal seam. The coal seam itself is both a source rock and a coalbed methane reservoir, with low permeability, mainly long-flame coal, and a small amount of gas coal in the deep. Affected by the conditions of the coal body, the critical gas pressures when coal and gas outbursts occur are slightly different. It can be seen from Fig. 7 that the outburst critical gas pressures of the two coal bodies varies logarithmically with the simulation depth. When the depth is shallow, the critical gas pressures of the two are similar, and there is no significant difference, indicating that in the shallow part of the mine, the difference of mechanical properties of the coal body has a low effect on outburst. After entering deep mining, under the action of high in-situ stress, the critical gas pressures of different coal samples differed significantly, indicating that with the increase of depth, the influence weight of mechanical properties of coal body on coal and gas outburst increases.

However, no matter what kind of coal sample, the outburst critical gas pressure presents a logarithmic variation rule with the depth. As the depth increase, the change amplitude of critical gas pressure gradually decreases, indicating that in deep mines, coal and gas outburst accidents are more sensitive to the effect of gas pressure. A slightly increase can lead to the occurrence of coal and gas outburst, and the outburst incubation time is significantly shorter than that of the shallow part, which brings great difficulty to the early predict and prevent of outbursts. The results of this test are like the previous results, which can explain the feasibility of this test from the side.

With the increase of depth, the maximum horizontal in-situ stress, minimum horizontal in-situ stress and vertical in-situ stress of coal seam increase accordingly. Therefore, the effect of in-situ stress in a single direction on the critical gas pressure is not considered. Instead, the effective stress and lateral pressure coefficient in-situ stress are used to comprehensively analyze the variation rule of the critical gas pressure. The effective stress $\sigma_{ea}$ can be calculated as follows:

2 $$\sigma_{ea} = \frac{1}{3}\left( {\sigma_{H} + \sigma_{h} + \sigma_{v} } \right) - \frac{1}{2}\left( {P_{cr} - P_{0} } \right)$$

where $\sigma_{ea}$ is the effective stress (MPa); $P_{cr}$ is the critical gas pressure (MPa); $P_{0}$ is the standard atmospheric pressure, 0.1 MPa.

The calculation formula of the lateral pressure coefficient in-situ stress γ is:

3 $$\gamma = \frac{{\sigma_{H} + \sigma_{h} }}{{2\sigma_{v} }}$$

where, *γ* is the lateral pressure coefficient of in-situ stress, dimensionless.

**Figure 8:**
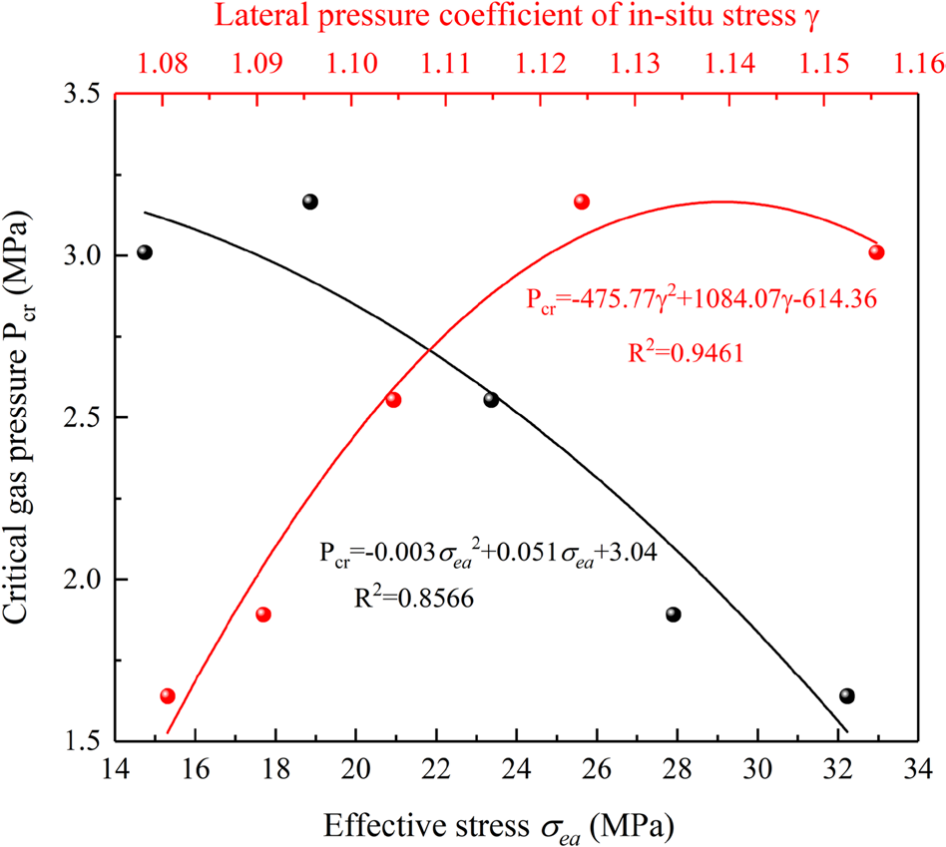
Relationship curves of effective stress, lateral pressure coefficient of in-situ stress and critical gas pressure.

The variation of critical gas pressure with effective stress and lateral pressure coefficient in-situ stress is shown in Fig. 8. The critical gas pressure decreases with the increase of the effective stress, showing a quadratic negative growth relationship. Under high in-situ stress, the coal and gas outburst can be triggered by applying a small gas pressure, which indicates the important role of in-situ stress in the process of outburst. The critical gas pressure increases with the increase of the lateral pressure coefficient in-situ stress, showing a quadratic growth relationship. The lateral pressure coefficient in-situ stress has a better correlation with the critical gas pressure than the effective stress. Therefore, in the future discussion of outbursts, more emphasis should be placed on the role of the lateral pressure coefficient in-situ stress. According to the theory of comprehensive action hypothesis, in-depth study on the effect of lateral pressure coefficient in-situ stress in an outburst can provide reliable support and reference for future research and field measurements.

### Analysis of gas pressure evolution characteristics

The process of coal and gas outburst can be divided into four stages: incubation, excitation, development and termination^51^. Under different depths, the gas pressure has three special variation rules during the incubation and excitation of coal and gas outbursts: (1) In the incubation, the gas pressure has the characteristics of self-increasing; (2) During the excitation, the gas pressure has the characteristics of stage and instantaneous. It is of great significance to study the evolution of gas pressure for the prevention and prediction of coal and gas outbursts.

#### Self-increasing characteristic of gas pressure

During the test, as the gas pressure gradually increased, the briquette specimens were gradually destroyed, resulting in many cracks. The gas content in the new cracks was low, which promoted the desorption of the gas adsorbed on the coal surface and in the pores, the gas pressure increased, the destruction of the coal accelerated, and causes coal and gas outbursts.

**Figure 9:**
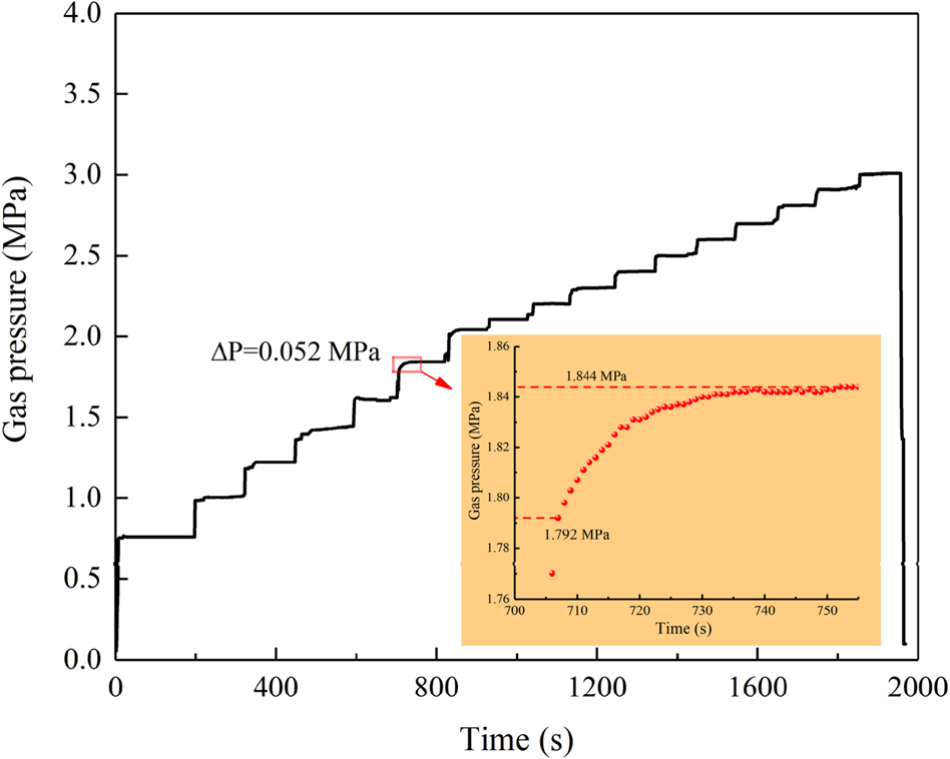
Evolution of gas pressure at 600 m.

After each stage loading, the gas pressure is still self-increasing slowly, this is due to the pre-adsorption the briquette before the outburst test, which made the briquette specimen reach the saturated adsorption state. Under the action of in-situ stress, the coal body was initially destroyed, and the type I crack was generated inside the coal body, providing a prerequisite for outburst. As the gas pressure is gradually increased, the destruction of coal body is aggravated. During the test, many new micro-cracks are generated in the coal body. The low content of gas in the new cracks makes the adsorbed gas change to free state and desorption phenomenon occurs. Since the test chamber is a closed space, the gas pressure will increase slightly at each gas pressure loading stage, although the loading is stopped. Test data under simulation depth of 600 m is described as an example, as shown in Fig. 9.

**Figure 10:**
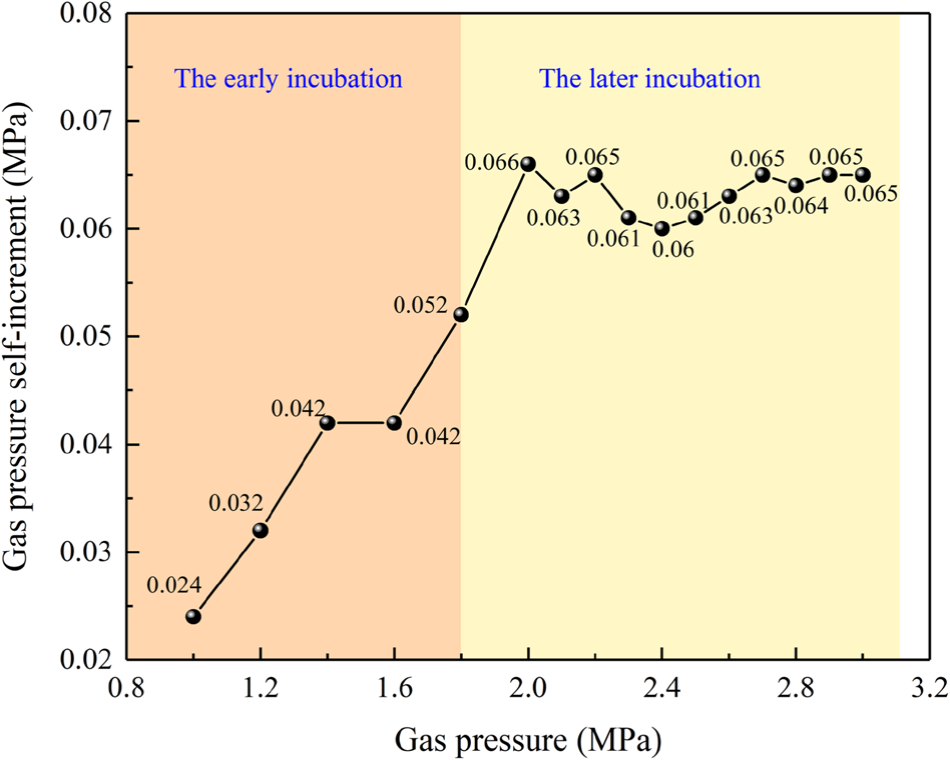
Gas pressure self-increment in each loading stage.

As can be seen from Fig. 9, the test proceeds to 707 s, where the gas pressure is loaded to 1.792 MPa, but the loading is stopped, the gas pressure has a slow self-increasing process (enlarged part of Fig. 9), gas pressure increase *ΔP* = 0.052 MPa. In each stage before, gas pressure has increased, but the increase is not obvious. It is due to the initial stage of gas pressure loading, the internal damage of the coal body is slow, and there are few newly cracks, the gas content of the desorption is low. From 1.792 MPa, there is an obvious self—increasing phenomenon, and each loading stage has an obvious increase process, with similar increase amplitude of 0.063 ± 0.003 MPa. During the whole loading process, the change of the gas pressure increment *ΔP* in each stage is shown in Fig. 10.

Under the simulation depth of 600 m, at the initial stage of gas pressure loading (0.75–1.8 MPa), the effective stress is relatively small, no large-scale damage occurs, only a few cracks are generated, the gas desorption amount is small, and the gas pressure increment not obvious, *ΔP* = 0.024, 0.032, 0.042, 0.042 MPa. When the gas pressure was loaded to 1.8 MPa, the specimen began to be damaged in a large area under the combined action of gas pressure and in-situ stress, resulting in many cracks, which promoted the rapid desorption of gas, and the gas pressure increased significantly, *ΔP* = 0.052 MPa. When the gas pressure reaches 2 MPa, *ΔP* is greatly raised to 0.066 MPa, and thereafter, each stage is floating on 0.063 MPa.

#### Stage and instantaneous characteristics of gas pressure

**Figure 11:**
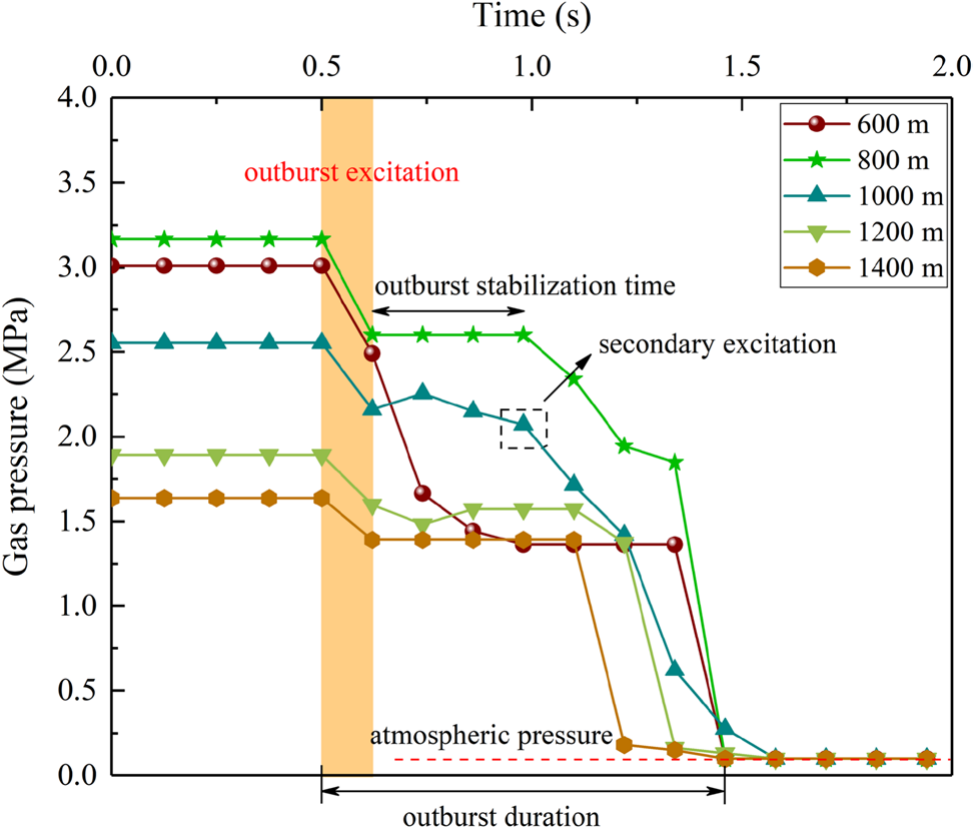
Variation of instantaneous gas pressure under different simulation depth.

In-situ stress and gas pressure loading are the process of providing energy for coal and gas outburst. When the critical gas pressure is reached, the energy accumulated in the coal body is rapidly released, and the coal is deformed and destroyed. After outburst excitation, gas pressure will further pulverize and throw coal. In this test, the in-situ stress mainly acted on the incubation and excitation. After the initiation of outburst, the destroy and ejection of coal body are mainly provided by gas pressure. Therefore, the test focuses on the variation characteristics of the instantaneous gas pressure of outburst. Figure 11 is the variation curve of gas pressure at the instantaneous of outburst under different simulation depths. To intuitively compare the variation of gas pressure, the five points before outburst were taken as the origin of outburst to analyze the variation rule of gas pressure at the instantaneous of outburst.

Taking the simulation depth of 1000 m as an example, when coal and gas outburst occurs, the gas pressure plummets rapidly from 2.554 to 2.164 MPa, and then slowly rises to 2.268 MPa, and the gas pressure is maintained at 2.164 MPa about 0.36 s. Secondary excitation occurred in the next 0.48 s, and the gas pressure rapidly dropped to 0.12 MPa, gradually approached atmospheric pressure. At this time, the outburst process basically ended, and the outburst duration is about 0.96 s. It can be seen from the variation process of gas pressure that the gas pressure decreases are 15.3%, 3.0% and 97.8% respectively, indicating that coal and gas outburst is not continuous, but a process of re-excitation after a short pause This is because the variation of the stress state in the chamber leads to the failure and instability of the coal body. When the outburst critical condition is reached, the elastic potential energy and gas internal energy accumulated in the coal body are released rapidly, and the broken coal body is thrown out. Due to the accumulation of partially broken coal at the outburst mouth, the gas-pulverized coal flow channel is reduced, and the gas pressure in the chamber will increase. When the new critical condition is reached, the coal body near the hole wall will be crushed and thrown out again. As the gas pressure gradually decreases, the outburst intensity will gradually weaken, it is difficult to meet the outburst conditions, and the outburst stop.

They all decrease to a certain pressure value and then stabilize for a period, and then decrease rapidly until it approaches atmospheric pressure. At this time, the outburst is finished. It indicates that the process of coal and gas outburst is not continuous, but after a short pause, the critical condition of outburst is reached again to induce outburst, forming secondary or even multiple outbursts. For example, three times of outburst occurred under the simulation depth of 800 m. This is because the critical gas pressure is the highest at 800 m. Therefore, the thrown coal mass will trigger multiple outbursts under the action of the gas pressure gradient, and its outburst intensity is the most serious.

According to the above analysis, in the process of outburst excitation, the gas pressure variations in stages, but with the increase of simulation depth, the less obvious in stages, the outburst duration decreases, and the outburst is more instantaneous. After outburst occurs, the gas pressure plummets, but not directly to atmospheric pressure. Instead, after a short period of constant pressure during the decline, it plummets again to atmospheric pressure. The entire instantaneous gas pressure experiences two or three times of pressure plummet processes, indicating that in excitation, gas pressure variation in stage.

### Analysis of influence of gas pressure on AE signal

The purpose of AE monitoring is to discover AE sources and obtain as much information about AE sources as possible. By processing and analyzing the detected AE signals, a large amount of information about deformation or failure in coal can be obtained, and the purpose of predicting the dynamic process of coal and gas outburst can be achieved. The generation and weakness of AE signal are closely related to the gas pressure loading process. The gas pressure loading leads to the damage of coal body and generates AE signal, which reflects the damage of coal body through AE signal. Therefore, it is reasonable and feasible to obtain precursor information of coal and gas outburst based on the change of AE signal.

#### Influence of gas pressure on AE energy frequency

The average AE energy under each loading pressure is calculated separately, the purpose is to analyze the degree of coal damage under different gas pressures, and to give the information indicators of coal and gas outburst precursors.

According to the test results, the AE energy amplitude E < 1000 mv∙ms is defined as the low energy, and the number of occurrences *N*_L_ is defined as the low energy frequency. The AE energy amplitude E ≥ 1000 mv∙ms is defined as the high energy, and the number of occurrences *N*_H_ is defined as the high energy frequency.

**Figure 12:**
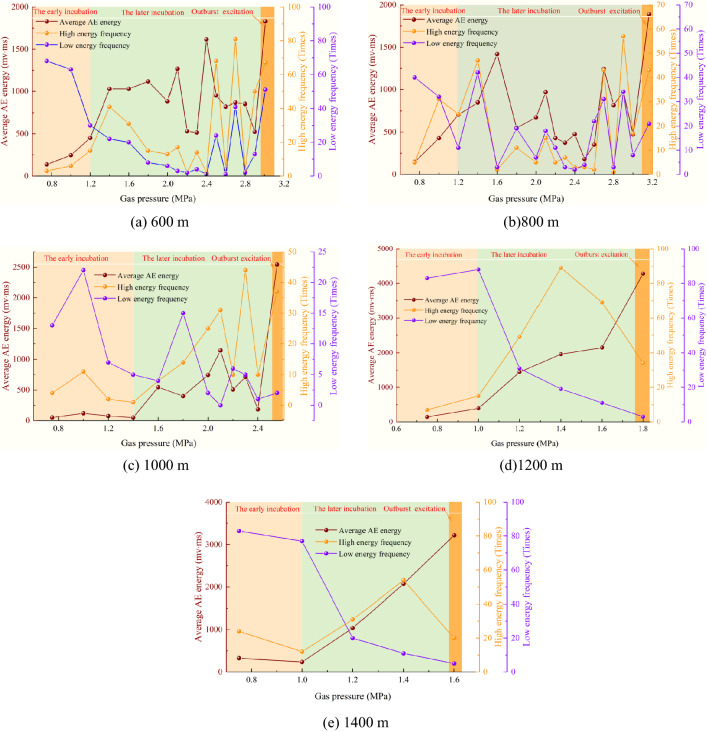
Curve of relationship among gas pressure, average AE energy and energy frequency.

The average AE energy, energy frequency and gas pressure variation under different simulation depths were calculated separately, as shown in Fig. 12, and the obtained rules are as follows:

(1) When the simulation depth is 600–1000 m, with the continuous loading of gas pressure, the average AE energy increases significantly in the later incubation and begins to have outburst risk. The high and low energy frequency variations actively and remains at a relatively high level, which means that the damage degree of the coal and rock mass reaches the limit, the danger of coal and gas outburst increases, and the coal mass is about to become unstable. At this time, it is the accumulation of energy. Once the gas pressure rises, the gas internal energy increases, reaching the energy destruction condition. The broken coal body is thrown out of the roadway with the gas from the weak face of the outburst. When the simulation depth is 1200–1400 m, with the continuous loading of gas pressure, the average AE energy continued to increase, the low energy frequency shows a downward trend, and the high energy frequency increased. However, the high energy frequency decreased from the later incubation to the excitation, but the high energy frequency is obviously higher than that of low energy frequency.

(2) With the loading of gas pressure, the variation trend of AE energy frequency of coal under low in-situ stress is fluctuating. When loading to 1.2 MPa, although the low energy frequency is close to the high energy frequency at 600 m, the high energy frequency is still dominant, indicating that the propagation of macro-cracks and the initiation of micro-cracks occurred simultaneously at this stage, and the damage degree of the coal body is severe. However, at 800 m, when loading to 2–2.5 MPa, the low energy frequency exceeds that of high energy frequency, and the low energy frequency dominant, and only micro-cracks are generated at this stage. It is because the micro-cracks provide new channels for gas seepage through laps and penetrations. When the crack is connected with others, the performance of the cracks is relatively smooth overall. At this time, the cracks are in the adjustment period, and only when the gas pressure continues to increase, the micro-cracks can continue to expand into macro-cracks. The results show that under the condition of deeper, due to the high level of in-situ stress on the coal body, the initial damage of coal body is serious. With the loading of gas pressure, the macro-cracks are more easily formed by the connection between the newly expanded micro-cracks and the original cracks. Therefore, coal and gas outburst are more likely to occur under the condition of high in-situ stress.

#### Determination of AE energy index of outburst dangerous

According to the variation characteristics of the average AE energy and energy frequency of coal and gas outburst at each stage under different simulation depths, the corresponding relationship between AE energy signal and precursor information of coal and gas outburst is established. The ratio of the average AE energy in the later incubation and the early incubation is defined as *L*_1_, and the ratio of the high energy frequency to the low energy frequency is defined as *L*_2_, then:

4 $$L_{1} = \frac{{\overline{{E_{2} }} }}{{\overline{{E_{1} }} }}$$

5 $$L_{2} = \frac{{N_{H} }}{{N_{L} }}$$

where $\overline{E}_{1}$ is the average AE energy in the early incubation (mv∙ms); $\overline{E}_{2}$ is the average AE energy in the later incubation (mv∙ms). The results are shown in Tables 3 and 4.

**Table 3:** Calculation results of *L*_1_ and *L*_2_ under different simulation depths.

Simulation depth(m)	${\overline{\text{E}}}_{{1}}$(mv∙ms)	${\overline{\text{E}}}_{{2}}$(mv∙ms)	*L*_1_	The early incubation			The later incubation		
				*N*_L_	*N*_H_	*L*_2_	*N*_L_	*N*_H_	*L*_2_
600	187	940	5.0	20	6	0.3	6	15	2.5
800	130	682	5.2	11	3	0.27	6	13	2.2
1000	67	605	9.0	10	2	0.2	4	14	3.5
1200	267	1872	7.01	27	4	0.15	11	41	3.7
1400	289	1611	5.5	24	6	0.25	12	30	2.5

**Table 4:** Risk index of coal and gas outburst.

*L*_*1*_	*L*_2_	Outburst risk	Energy frequency
< 5.0	< 0.3	Weak	Low energy frequency dominant
> 5.0	> 2.2	Strong	High energy frequency dominant

Through the statistics of the acoustic emission indicators at different simulation depths at each stage, combined with the previous analysis of the risk of AE energy outburst early and later incubation. The critical index of coal and gas outburst risk under the test conditions in this paper are determined, as shown in Table 4. However, due to the limitation of test conditions, the universality of the application of outburst risk index needs further research.

1. When *L*_1_ < 5.0, *L*_2_ < 0.3, the low energy frequency is higher than that of high energy frequency, low AE energy is dominant, only micro-cracks occur in the coal body, and it is in the early incubation, with a weak outburst risk.
2. When *L*_1_ > 5.0, *L*_2_ > 2.2, the high energy frequency is higher than the low energy frequency, high AE energy is dominant, and the cracks changes from micro to macro in the coal body, which is in the later incubation with a strong outburst risk.

## Discussion

Coal is a porous medium with strong adsorption^52,53^. Inside coal, micropores with pore radius less than 40 nm account for about 90% of the total pore volume, and the specific surface area can reach 200 m^2^/g^54^. The large specific surface area of coal creates conditions for gas adsorption. In coal seams, when external conditions such as gas pressure, temperature, etc., are constant, gas adsorption and desorption transformation is always in a dynamic equilibrium state. When the free gas concentration in the pores decreases, the gas adsorbed on the pore surface is released into the pores and transformed into free gas.

During the incubation of coal and gas outburst, under the action of stress, the internal damage of a coal mass continues to develop, resulting in the continuous generation, growth, and expansion of microcracks^55^. The low concentration of free gas in the new cracks promotes the transformation of adsorbed gas to free gas, increases the gas pressure in the cracks, promotes the further development of cracks, and leads to further destruction of coal body. Jiang and Yu^56^ proposed the spherical shell instability hypothesis of gas-bearing coal failure through hypothesis demonstration and derivation. That is, when the coal and gas outburst occur in rock cross-cut coal uncovering, the coal body in front of uncovering coal is destroyed under the action of a certain dynamic stress field, the coal body and gas continuously pour into the roadway, and the outburst front quickly penetrates the coal seam.

**Figure 13:**
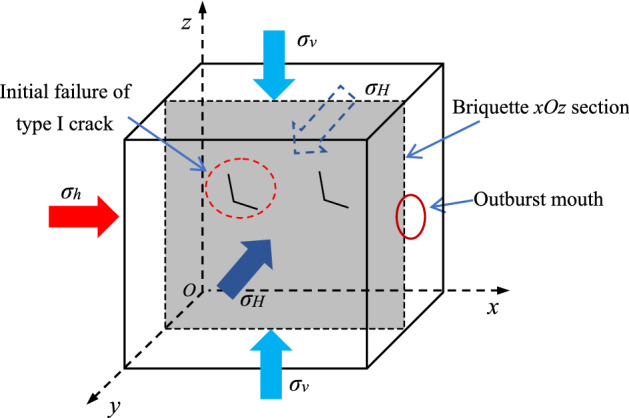
Initial failure of gas-bearing coal.

In this paper, the test is conducted in a 200 mm × 200 mm × 200 mm closed pressure chamber. The in-situ stress remained constant, and the gas pressure increased in a stepwise. The failure criterion of gas-bearing coal body is slightly different. Analysis of any xOz section of the briquette specimen, assuming that the section contains the I-type cracks generated after the initial action of in-situ stress, and the coal particles and pores in the section reach saturation adsorption state. The force analysis of the section is shown in Fig. 13. At the beginning of gas pressure loading, the coal body is in the stage of in-situ stress failure, that is, the initial failure of the gas-bearing coal body. In the coal seam, the initial failure of the gas-bearing coal body is completed by the in-situ stress. After the initial action of in-situ stress, type I cracks are generated, which provides the preconditions for outburst.

**Figure 14:**
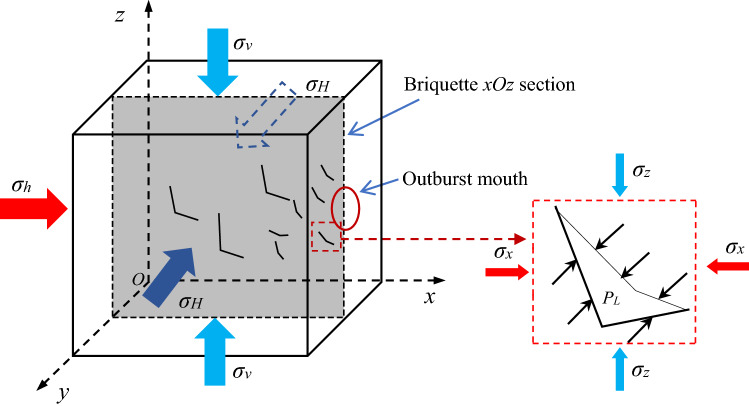
Force analysis of crack propagation in gas-bearing coal body.

With the continuous loading of gas pressure, the coal body enters the gas tearing the coal body stage, as shown in Fig. 14. In this stage, with the increase of gas pressure, many new cracks are generated inside the coal under the combined action of in-situ stress and gas pressure. The gas concentration in the new crack is low, and the adsorbed gas near and on the surface of the crack channel is desorbed and released into the cracks, and the gas is desorbed toward the surface of the channel. When the stress on the cracks in the coal body and the gas pressure in the cracks meet the crack propagation conditions, the gas pressure will stretch the cracks and further expand the cracks. After the propagation, the gas concentration in the crack decreases again, and the gas adsorbed on the crack surface continues to desorb, releasing gas into the crack and repeating the crack propagation process. The micro-cracks continue to expand, forming a network of cross-cracks or connecting with type I cracks to form large cracks. Under the action of gas pressure, the crack network continues to extend to the outburst mouth. Before the outburst occurs, the coal body structure is completely destroyed, skeleton is unstable, the gas pressure continues to rise and reaches the critical value of the outburst. Finally, the weak surface is damaged, and the outburst occurs.

Take a micro unit containing cracks in the coal body for force analysis. It is assumed that the selected micro unit contain not only enough pores and cracks which can reflect the structural characteristics of coal body, but also enough adsorbed gas. The outside of the crack is compressed by the surrounding coal, and the inside of the crack is expanded by gas pressure. When the compressive stress $\sigma_{i}$ ($\sigma_{i}$ is the resultant force of $\sigma_{x}$ and $\sigma_{z}$) and the gas tension force *P*_*L*_ meet the crack propagation criterion, the gas exerts a tensile expansion effect on the crack. According to the theory of fracture mechanics, the crack propagation conditions are:

6 $$K_{I} = K_{IC}$$

where *K*_*I*_ is the stress intensity factor, which is a parameter to measure the intensity of the stress field at the crack tip (MPa∙m^0.5^); *K*_*IC*_ is the fracture toughness of the material, which is an index of the material's resistance to fracture, can be measured by experiments (MPa∙m^0.5^).

When the stress intensity factor at the crack tip is equal to the fracture toughness of the material, the crack begins to propagate. The stress intensity factor can be expressed as:

7 $$K_{I} = Y\sigma_{I} \sqrt {\frac{{{\uppi }a}}{2}}$$

where *Y* is the crack shape coefficient, which depends on the crack type; $\sigma_{I}$ is the tensile stress of the crack (MPa); a is the crack size (m).

When $Y\sigma_{I} \sqrt {\frac{\pi a}{2}} = K_{IC}$, the crack propagation condition is satisfied, then the crack tensile stress needs to satisfy:

8 $$\sigma_{I} = \frac{{\sqrt 2 K_{IC} }}{{Y\sqrt {\pi a} }}$$

According to the force analysis of coal fracture unit in Fig. 14, the tensile stress ${\upsigma }_{{\text{I}}}$ of coal fracture can be expressed as:

9 $$\sigma_{I} = P_{L} - \sigma_{i}$$

where *P*_*L*_ is the gas pressure in the fracture (MPa); $\sigma_{i}$ is the resultant of compressive stress on the fracture (MPa).

Substituted Eq. (9) into Eq. (8), under this test conditions, the criterion of coal crack propagation under gas pressure is:

10 $$P_{L} - \sigma_{i} = \frac{{\sqrt 2 K_{IC} }}{{Y\sqrt {\pi a} }}$$

When the gas pressure in the fracture meets this condition, the fracture begin to propagation. With the propagation of fracture in the coal body, that is, the increase of a, the gas pressure required for fracture propagation in the coal body gradually decreases. Therefore, after coal and gas outburst, fractures occur in the coal body. The existence of gas makes the fractures to continue to propagation to the deep, resulting in a gradual decline in the gas pressure of the deep^57^.

As discussed above, after the outburst occurred, the coal body near the outburst mouth is violently destroyed under the action of gas pressure and in-situ stress, and then thrown into the mining space, the gas pressure dropped sharply. With the development of cracks in coal to the depth of coal seam, the gas pressure in the deep coal body decreases slowly. Simultaneously, the existence of gas reduces the effective stress and shear intensity of the coal body^58^, which accelerates the tensile shear failure of the coal body. Moreover, the cracks in the coal body can propagate to the deep under a small gas pressure gradient and provide a channel for the gas flow in the deep coal body.

## Conclusions

In this study, the self-developed device is used to conduct simulation experiments at different simulation depths. Combined with acoustic emission equipment, the evolution of the whole outburst process and the instantaneous gas pressure are explored. The main conclusions are as follows:

1. Gas pressure not only provides the throwing kinetic energy of coal outburst, but also can accelerate the destruction of the coal by the gas adsorption and desorption characteristics during the incubation.
2. With the increase of depth, the critical gas pressure of outburst gradually decreases, the unit outburst intensity gradually increases. The gas threshold value of deep coal and gas outburst is low, and gas is the main energy source of outburst.
3. During the incubation and excitation of coal and gas outbursts, gas pressure has three special variation rules, namely self-increasing characteristic, stage and instantaneous. The outburst process is usually not continuous, but after a short pause, the critical condition of outburst is reached again to induce outburst, forming secondary or even multiple outbursts.
4. From the perspective of AE energy, the quantitative index reflecting the risk of coal and gas outburst in the incubation is defined. When *L*_1_ < 5.0, *L*_2_ < 0.3, it is in the early incubation with a weak outburst risk. When *L*_1_ > 5.0, *L*_2_ > 2.2, it is in the later incubation with a strong outburst risk.

## Data Availability

The datasets generated and/or analysed during the current study are not publicly available due ongoing further experiments but are available from the corresponding author on reasonable request.
